# Sevoflurane Anesthesia Improves Cognitive Performance in Mice, but Does Not Influence *In Vitro* Long-Term Potentation in Hippocampus CA1 Stratum Radiatum

**DOI:** 10.1371/journal.pone.0064732

**Published:** 2013-05-28

**Authors:** Rainer Haseneder, Laura Starker, Jasmin Berkmann, Kristine Kellermann, Bettina Jungwirth, Manfred Blobner, Matthias Eder, Eberhard Kochs, Gerhard Rammes

**Affiliations:** 1 Department of Anesthesiology, Klinikum rechts der Isar, Technische Universität München, Munich, Germany; 2 Research Group Neuronal Network Dynamics, Max Planck Institute of Psychiatry, Munich, Germany; Univ. Kentucky, United States of America

## Abstract

**Background:**

Whether the occurrence of postoperative cognitive dysfunction is a result of the effects of surgery or anesthesia is under debate. In this study, we investigated the impact of sevoflurane anesthesia on cognitive performance and cellular mechanisms involved in learning and memory.

**Methods:**

Male C57Bl6/J mice (4–5 months) were exposed to one minimum alveolar concentration sevoflurane for two hours. After 24 h, cognitive performance of mice was assessed using the modified hole board test. Additionally, we evaluated hippocampal long-term potentiation and expression levels of different receptor subunits by recording excitatory postsynaptic field potentials and using the western blot technique, respectively. Non-anesthetized mice served as controls.

**Results:**

In anesthetized mice, neither cognitive performance nor long-term potentiation was impaired 24 h after anesthesia. Interestingly, sevoflurane anesthesia induced even an improvement of cognitive performance and an elevation of the expression levels of *N*-methyl-D-aspartate (NMDA) receptor type 1 and 2B subunits in the hippocampus.

**Conclusions:**

Since NMDA receptor type 1 and 2B subunits play a crucial role in processes related to learning and memory, we hypothesize that sevoflurane-induced changes in NMDA receptor subunit composition might cause hippocampus-dependent cognitive improvement. The data of the present study are in favor of a minor role of anesthesia in mediating postoperative cognitive dysfunction.

## Introduction

Postoperative cognitive dysfunction (POCD) is a decline in cognitive performance for weeks or months which occurs after surgery. Although there is general agreement that POCD is likely to be multifactorial, it remains unclear, whether its occurrence is a result of anesthesia, surgery or a combination of both [1].

Animal studies investigating the impact of general anesthesia on memory formation are rather inconsistent, and showed both an impairment or improvement of cognitive function. Acquisition of new memory and performance improvement in an already-learned spatial memory task was impaired in aged rats up to two weeks after isoflurane/nitrous oxide anesthesia [2], [3]. Adult rats showed an impaired acquisition of new memory only when tested two days, but not two weeks after isoflurane/nitrous oxide anesthesia, when spatial memory performance was even improved [4], [5]. When 12-month-old mice were repetitively exposed to isoflurane, a decrease in cognitive performance was observed, which did not occur, when the animals were exposed to halothane [6]. Other studies showed an enhancement of some aspects of learning and memory, when animals were exposed to low concentrations of volatile anesthetics [7]–[9], or when rats where exposed to isoflurane during fetal stage [10]. We could show recently, that isoflurane anesthesia improves cognitive performance, enhances hippocampal long-term potentiation (LTP) and modulates the expression levels of *N*-methyl-D-aspartate (NMDA) receptor subunits [11].

The molecular mechanisms of the commonly used volatile anesthetic sevoflurane are still a matter of debate. Sevoflurane potentiates γ-aminobutyric acid type A (GABA_A_) and glycine receptor function, and inhibits nicotinic acetylcholine, α-amino-3-hydroxy-5-methyl-4-isoxazolepropionic acid (AMPA), and NMDA receptor function (for review see: [12]). In hippocampal slice preparations, sevoflurane depresses synaptic transmission to CA1 pyramidal neurons [13], [14] at least in part by an activation of GABA_A_ receptors [14]–[16].

LTP is an enhancement of synaptic efficiency upon repetitive and/or simultaneous stimulation of afferent inputs and represents an important and well studied form of synaptic plasticity. It has been shown that volatile [17]–[19] and intravenous [20], [21] anesthetics abolish the formation of LTP when applied during the LTP-inducing stimulus. Since LTP is considered as one of the major cellular mechanisms that underlies learning and memory (for review see: [22]), it has been suggested that its blockade might contribute to POCD [17], [23].

There is data, that the occurrence of POCD might be agent-specific [6], [24], [25]. However, published data of studies using animal models investigating the impact of anesthesia on cognitive performance is largely limited to isoflurane or combined isoflurane/nitrous oxide. Therefore, in the study at hand, we investigated the impact of sevoflurane anesthesia on cognitive performance, synaptic plasticity and expression of neurotransmitter receptors in mice.

## Methods

### Animals

Male C57Bl6/J mice (Charles River, Sulzfeld, Germany) were investigated at the age of 4–5 months. All mice were housed separately under standard laboratory conditions (12:12 h light/dark cycle, 22°C, 60% humidity) and had free access to tap water and standard mouse chow. Prior to the investigations, mice were allowed to habituate to their new surroundings for at least three weeks after having been transferred from the breeder.

### Anesthesia

Mice were placed in an acrylic glass chamber (FiO_2_  = 0.4; T = 32°C). In the anesthesia group (sev) the chamber was pre-flushed with 5.0 vol% sevoflurane to induce anesthesia. After loss of postural reflexes, the induced animal was removed from the chamber. The non-anesthetized animals (sham) were replaced in their home cages after four minutes, whereas the nose of the anesthetized mouse was put in a continuously flushed chamber (3 l/min) with a moderately increased pressure (3 mmHg) to prevent pulmonary atelectasis. There, the mouse spontaneously breathed one minimum alveolar concentration (MAC) sevoflurane in air and oxygen (FiO_2_ = 0.5). Heart rate and respiratory frequency were monitored. Rectal temperature was maintained between 37–38°C by applying a warming blanket. To avoid influence of instrumentation on cognitive and behavioral testing, mice were not cannulated. After two hours of anesthesia, the animals were replaced in the induction chamber, which was then flushed with oxygen. After recovery, the mice were returned to their home cages.

### Cognitive testing

The modified hole board test (MHBT), which enables the investigation of cognitive, exploratory, and motivational parameters in rodents [26], was used to assess the cognitive performance of mice after sevoflurane anesthesia or sham treatment.

The MHBT consists of an opaque grey polyvinyl chloride board on which ten cylinders (3×3×3 cm) are placed in two lines. The board (35×18×1 cm) is placed in the middle of a polyvinyl chloride box (50×50×50 cm), thus representing the unprotected central area of an open field. The outer protected area is divided into eight quadrants (16×16 cm) by white lines. Each cylinder contains a small piece of almond (0.05 g), which is placed underneath a grid and, therefore, cannot be removed by the animals. In addition, each cylinder is flavored with vanilla essence (dissolved in water 0.02%; Micro-Plus, Stadtoldendorf, Germany), as mice are attracted to this flavor. Three of the ten cylinders are marked with white tape and in these, another piece of almond is placed on top of the grid as a food reward, which can be reached by the animals. The positions of these marked and baited cylinders on the board were changed randomly. Mice should learn that only marked cylinders are baited with food.

According to previous studies [26]–[28], several cognitive functions were evaluated. A hole was counted as visited if the animal poked its nose over the rim of the cylinder. *Omission errors* were defined as the number of marked and baited holes, which were not visited at all during one trial. *Wrong choices* represents the number of non-marked holes, which were visited. The time each animal required for performing the trial (i.e. visiting all three baited holes; max. 5 min; *time trial*) indicates cognitive performance as well as the motivation to perform the test.

Additionally to the assessment of the described cognitive parameters, the MHBT also allows an investigation of a variety of behavioral parameters that can be assigned to ethologically given and previously validated behavioral categories [26]–[28]. The number of times the mouse enters the board (*board entries*) served as an indicator for anxiety. The number of times the mice crossed the marked lines per minute (*line crossings*) was counted and assessed as an indicator for locomotor activity.

In the experimental set-up, all mice were placed one after the other on the starting position in the experimental box and were allowed to explore the box freely for a maximum of five minutes or until they found and ate all three food rewards. Three days before anesthesia, each mouse was habituated to the food reward by receiving pieces of almond in their home cage. The test was performed on eight consecutive days (D1 to D8). The first examination took place 24 h (D1) after anesthesia or sham treatment. Four trials were carried out per day, with one trial lasting for a maximum of 300 s followed by a minimum of ten minutes inter-trial interval.

Compared to other cognitive tests, e.g. the Morris water maze or the radial arm maze, the MHBT provides several advantages [29]. First, by supplying a highly attractive food-reward, the mice do not have to be deprived of food or water, which potentially could induce stress. Furthermore, they are motivated to perform the test voluntarily, and this also has been shown to avoid test inherent stress. Second, a variety of behavioral parameters can be evaluated in addition to cognitive parameters in one session, allowing for the assessment of altered motivational systems, which could potentially have an impact on cognitive performance. A recently published direct comparison showed that the MHBT is at least not inferior compared to the Morris water maze in assessing the functional outcome of rats in a model of cerebral ischemia [30].

### Electrophysiology

Sagittal hippocampal brain slices (350 µm thick) were obtained from mice 24 h after anesthesia or sham treatment. After killing an animal by cervical dislocation, the brain was rapidly removed and slices were prepared in ice-cold artificial cerebrospinal fluid (ACSF) using a vibroslicer (HM 650 V, Microm International, Walldorf, Germany). Slices were allowed to recover for at least 1 h before being transferred to the recording chamber which was continuously perfused with ACSF at a rate of 5 ml/min. The ACSF contained (in mM): NaCl, 125; KCl, 2.5; NaHCO_3_, 25; CaCl_2_, 2; MgCl_2_, 1; D-glucose, 25; NaH_2_PO_4_, 1.25 (all substances from Sigma, Deisenhofen, Germany). Saturation with a mixture of 95% O_2_/5% CO_2_ (carbogen gas) led to a pH of 7.4. All experiments were performed at room temperature (22–24°C). Extracellular recordings of field excitatory postsynaptic potentials (fEPSPs) were performed in the CA1 stratum radiatum of the hippocampus using borosilicate glass micropipettes (1–2 MΩ) filled with ACSF. Data were recorded with an Axopatch 200B patch-clamp amplifier, a Digidata 1200 interface (both from Axon Instruments, Foster City, CA), and the LTP Program 2.30d software [31]. fEPSPs were evoked by electrical stimulation of the Schaffer collateral/associational commissural pathway with a bipolar tungsten electrode insulated to the tip (50 µm tip diameter). Stimuli were delivered at 30 s intervals, and two consecutive fEPSPs were averaged for noise minimization. The initial slope of the rising phase of the fEPSP (taken between 20% and 80% of the peak amplitude) was used as measure of the strength of synaptic transmission. For baseline recordings, stimulation intensity was set to a value that evoked a response approximately 25–30% of the maximal inducible response. After a minimum of 30 min of stable baseline recordings, a high frequency stimulus (HFS; 100 pulses delivered at 100 Hz) was applied to the Schaffer collateral/associational commissural pathway. After HFS, recordings were made for 40 min without changing the rate or intensity of the stimulus, and fEPSP slopes were normalized with respect to the responses recorded during the last 5 min before HFS. LTP, quantified as percental elevation of fEPSP slopes 36–40 min after HFS, was compared between brain slices of mice that underwent sevoflurane anesthesia or sham treatment.

### Analysis of Receptor Expression

24 h after anesthesia or sham treatment, mice were killed by cervical dislocation, decapitated and their brains were rapidly removed. Brains were immediately frozen on dry ice. Subsequently, the hippocampus was dissected and kept at −80°C until used for Western blotting.

The hippocampus of each animal was homogenized in HEPES buffer containing 1% NP40 and several proteinase inhibitors (based on [32]), and centrifuged to eliminate cell debris. The supernatant was used as total protein sample. Protein concentration was determined with the BioRad DC protein kit (BioRad, Munich, Germany). Protein samples (25 µg) of each animal (n = 6 per group) were loaded on 9% SDS–PAGE and transferred to nitrocellulose (Protran BA85, 45 µm, Schleicher and Schüll, Dassel, Germany), using a Mini Transfer Cell (BioRad, Munich, Germany). The membranes were blocked with 5% BSA in TBS containing 0.1% Tween 20 (TBS-T) and incubated with the different primary antibodies overnight. The following antibodies were used for Western blot analysis: NMDAR1, NMDAR2A, NMDAR2B, GluR1, GluR2/3, GluR4, GluR6/7, α_2_-GABA_A_, and β_2_-nAChR (all from Millipore, Schwalbach, Germany). Incubation with the secondary antibody (horseradish peroxidase-conjugated donkey anti-rabbit antibody, Amersham Buchler, Braunschweig, Germany) lasted two hours. All antibody incubations, washes and dilutions were performed in TBS-T. Antibody detection was performed with the Amersham ECL Western blotting analysis system according to the manufacturer's protocol. ECL signal was exposed to Hyperfilm-ECL (Amersham Buchler, Braunschweig, Germany). To verify equal loading of protein, the same nitrocellulose membrane was re-stained and the total amount of protein of each lane was assessed. Unless stated otherwise, all chemicals were obtained from Sigma (Deisenhofen, Germany).

At least three blots were prepared per antibody, which were analyzed and averaged. Each Western blot comprised the control and the remaining experimental group. Blot autoradiographs were quantified by computer-assisted densitometry using the Optimas image analysis system (BioScan Optimas, Edmonds, WA). All data are expressed as relative grey values and, for each subunit, the values for the anesthetized and sham group were determined by setting the sham group to 100% and calculating the relative percentages of the anesthetized group. The respective group values were pooled as mean ± SEM.

### Statistical Analysis

Statistics were performed using SPSS 14.0 for Windows (SPSS Inc., Chicago, IL). A two-tailed probability value of P<0.05 was considered as statistically significant. For analysis of cognitive and behavioral parameters, the data was assessed in a one- or two-factor ANOVA, respectively. Statistical analysis for electrophysiology was carried out using the Student’s t-test with a level of P<0.05 required for significance. Averaged values are given as mean ± SEM. Western blot data were proven for significance by comparing the grey values of the anesthetized and non-anesthetized group of each subunit separately using the Student’s t-test. Normal distribution of data was verified using the Kolmogorov-Smirnov test.

### Ethics Statement

All studies were approved by the Ethical Committee on Animal Care and Use of the Government of Bavaria, Germany (permit number: 55.2-1-54-2531-72-05). All efforts were made to minimize animal suffering and to reduce the number of animals used.

## Results

### Sevoflurane anesthesia improves cognitive performance in mice

**Figure 1:**
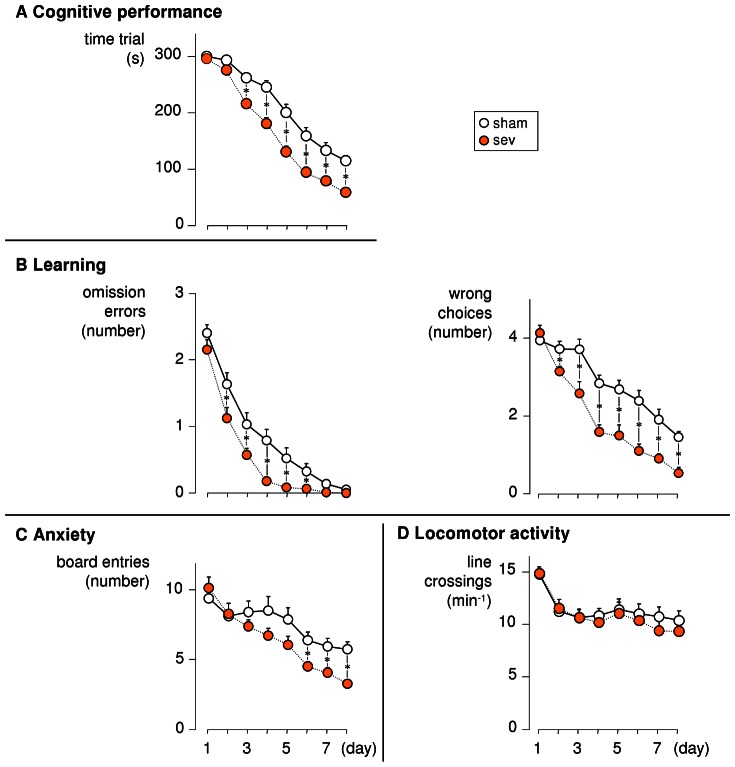
Mice that underwent a sevoflurane anesthesia showed better cognitive performance. On days one to eight after undergoing a sevoflurane anesthesia (sev) or sham treatment (sham), cognitive performance and behavorial parameters were assessed using the modified hole board test, a task in which the animals are trained to search for food rewards hidden in marked cylinders. **A**: Time that each animal required for performing the trial plotted against time. **B**: Number of marked and baited holes, which were not visited at all during one trial (left) and number of non-marked holes which were visited (right) plotted against time. **C**: Number of times the mouse enters the board plotted against time. **D**: Number of times the mouse crossed the marked lines per minute plotted against time. Each group consisted of 24 animals. Each symbol represents averaged data from four trials per day. * p<0.05 reveals better cognitive performance (beginning on day three) and better learning (beginning on day two), as well as an attenuated anxiety-related behavior (beginning on day six) in anesthetized mice.

To determine whether sevoflurane anesthesia without surgery affects learning and memory, various cognitive and behavioral parameters were studied using the MHBT. In Fig. 1, *time trial* (A), *omission errors* and *wrong choices* (B), *board entries* (C) and *line crossings* (D) are plotted against time. Substantial learning occurred in all groups, which could be proven by a one-factor ANOVA of each curve, showing a significant effect of time on *time trial*, *omission errors, wrong choices* and *board entries* (all P<0.001). Group comparisons revealed that, compared to non-anesthetized controls, anesthetized mice showed better overall cognitive performance (*time trial*) beginning on day three, and better degree of learning (*omission errors* and *wrong choices*) beginning on day two. Starting from day six, anesthetized showed a less anxious behavior (*board entries*).

### Sevoflurane anesthesia does not affect LTP after 24 h

**Figure 2:**
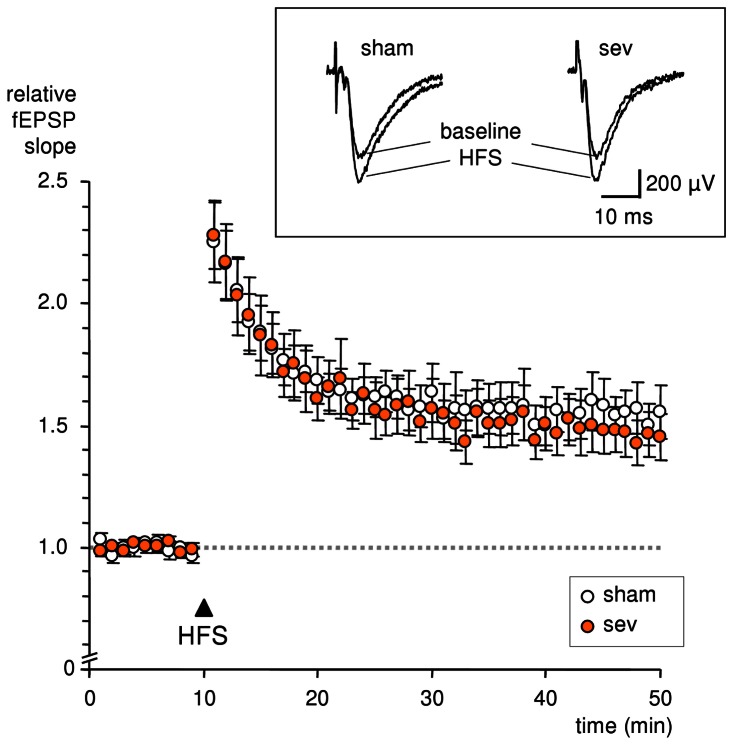
Long-term potentiation (LTP) in hippocampal brain slices of anesthetized and sham-treated mice was not different. 24 h after sevoflurane anesthesia (sev) or sham treatment (sham), brain slices of the animals were prepared and hippocampal LTP was assessed as elevation of field excitatory postsynaptic potential slopes (fEPSP slopes) after high frequency stimulation (HFS). HFS led to an LTP of fEPSP slopes, which was not significantly different between the two groups. Each symbol represents the averaged fEPSP slopes normalized with respect to the 5 min baseline period before HFS. Insets show fEPSP recordings before and 40 min after HFS.

To evaluate the possibility that sevoflurane anesthesia could affect synaptic plasticity, LTP was studied in hippocampal slices of anesthetized and non-anesthetized mice one day after anesthesia or sham treatment. Delivering HFS induced LTP of fEPSP slopes, which was not significantly different between slices of anesthetized (sev) or non-anesthetized (sham) mice (relative fEPSP slopes sev: 149.6±9.4% (n = 9); sham: 159.5±9.4% (n = 10; P = 0.48); Fig. 2).

### Expression of NMDA receptor subunit type 1 and 2B is upregulated after sevoflurane anesthesia

**Figure 3:**
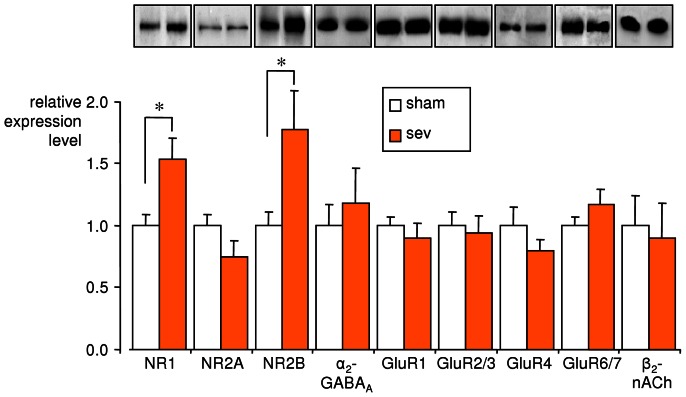
Sevoflurane anesthesia induced changes in the expression of *N*-methyl-D-aspartate (NMDA) receptor subunits in the hippocampus. Western blot analysis was used to determine the protein expression levels of various receptor subunits in the hippocampi of anesthetized (sev) and non-anesthetized (sham) mice. Changes in expression levels in the sev group are expressed as relative values normalized to the grey values of the sham group. *P<0.05 reveals an upregulation of the *N*-methyl-D-aspartate receptor type 1 and 2B subunits (NR1 and NR2B) after sevoflurane anesthesia. No other receptor subunit was significantly altered. Example immunoblots are depicted in the insets. NR2A  =  NMDA receptor type 2A subunit; α_2_-GABA_A_  = α_2_ subunit of γ-aminobutyric acid type A receptor; GluR1, GluR2/3, GluR4  = α-amino-3-hydroxy-5-methyl-4-isoxazolepropionic acid receptor subunits 1, 2/3, 4; GluR6/7 =  kainate receptor subunit 6/7, β_2_-nAch  = β_2_ subunit of nicotinic acetylcholine receptor.

Sevoflurane anesthesia might induce adaptational changes in the expression levels of neurotransmitter receptor subunits. We used western blotting for profiling the expression levels of the NMDA receptor type 1, type 2A and type 2B subunits (NR1, NR2A, and NR2B), subunits of α-amino-3-hydroxy-5-methyl-4-isoxazole­propionic acid (GluR1, GluR2/3, GluR4), kainate (GluR6/7), GABA_A_ (α_2_), and nicotinic acetylcholine (β_2_) receptors in hippocampi of anesthetized and non-anesthetized mice (Fig. 3). In homogenates of the hippocampi of anesthetized mice, we found an upregulation of the NR1 subunit (153±17% of control, n = 11, P = 0.01) and the NR2B subunit (177±31% of control, n = 11, P = 0.03). The expression levels of the other receptor subtypes did not change significantly (Fig. 3).

## Discussion

The aim of the present study was to determine medium-term effects of sevoflurane anesthesia on cognitive performance, LTP, and receptor subunit expression in mice. We found that an anesthesia with sevoflurane, applied at a clinically relevant concentration, improved cognitive performance, but had no effect on hippocampal LTP. The improved cognitive performance might be explained by the elevated expression levels of the NR1 and NR2B subunits in the hippocampus.

In the present study, we could see an improvement of hippocampus-dependent cognitive performance in animals that were anesthetized. This improvement has been observed starting from day two lasting until the end of the test at day eight. Additionally, anesthetized animals showed an attenuated anxiety behavior beginning at day six. Cognitive processes and anxiety are closely related and interacting processes. Since the attenuated anxiety developed considerably after the improvement of cognition, we interpret the reduced anxiety levels as consequence of the improved cognitive performance. Anesthetic-induced positive [7]–[11], [33] and negative [2]–[4], [6], [34] effects on cognition and memory formation in rodents have been reported. Obviously, memory effects of anesthetics in rodents do critically depend on test environment, age, and time between anesthesia and cognitive testing. The sevoflurane-induced improvement of cognitive performance seen in the present study is strictly in line with our recently published data, that a preceding isoflurane anesthesia improves cognitive function [11].

Additionally, we could show that sevoflurane anesthesia induces an elevation of the expression of the NMDA receptor NR1 and NR2B subunit in the hippocampus 24 h after the anesthesia. An altered expression of diverse genes in the hippocampus or amygdala has been shown in rats being anesthetized with isoflurane/nitrous oxide [35] or isoflurane alone [36]. On the protein level, it has been shown that desflurane induces an alteration of several intracellular proteins, which are important for the endocytosis of neurotransmitter receptors [37].

The NMDA receptor has been shown to be important for learning and memory [22], [38], and a critical role for the NR2B subunit in processes related to learning and memory has been shown by a number of studies. Genetic overexpression of the gene encoding NR2B led to mice with improved learning and memory in a variety of behavioral tasks [39], [40], whereas a hippocampal NR2B deficit impaired spatial learning [41]. Therefore, the described improvement in cognitive function after sevoflurane anesthesia, which was already detectable in the early phase of our behavioral testing, might be explained by the upregulation of the NR1 and NR2B subunits of the NMDA receptor. However, our experimental conditions do not allow the conclusion that the retained cognitive improvement is the result of a permanent up-regulation of NMDA subunits by sevoflurane. A very recent study reports an upregulation of NR1 and NR2B subunits of the NMDA receptor after 4 h of isoflurane/nitrous oxide anesthesia in 18-month-old rats, which was associated with an impaired spatial learning [34]. This discrepancy to our data can convincingly be explained by the age-dependent implications of NMDA receptor levels on memory: In older rats, high NR1 and NR2B levels correlate with a decline in memory, whereas in younger ones, high NR1 and NR2B correlate with an improvement [42].

There is evidence that an elevation of NR2B subunits results in enhanced LTP [39]. In the present study, we observed an elevated expression level of NR2B subunits in hippocampus homogenates without enhancement of LTP at the CA1 stratum radiatum synapse. One possible explanation might be that the observed upregulation of NR2B subunits occurs at synapses different from CA1 synapses (e.g. CA3, gyrus dentatus). Numerous reports show an improved cognitive performance and memory formation accompanied by an enhanced LTP in certain central nervous system regions (for review see e.g. [43]). However, this correlation must not necessarily be a general accepted principle (for reviews see e.g. [44], [45]). The lack of an *in vitro* and *in vivo* correlation of memory related processes might be explained by a hippocampus-specific improvement of cognitive performance due to altered neuronal activity in all hippocampal regions and/or in connected brain areas, which is consequently not detectable with recordings from the CA1 region only. For sevoflurane, a memory enhancing effect in rats has been described, which is dependent on the basolateral amygdala [8].

Data on the impact of anesthesia on synaptic plasticity is largely limited to studies describing the effect of acute, *in vitro* application of anesthetics during induction of LTP. To our knowledge, our previous study [11] is the only one that investigated the impact of a preceding anesthesia on synaptic plasticity so far. In this study, we showed an enhanced hippocampal LTP 24 h after an isoflurane anesthesia in mice. In the present study, the potentiation of synaptic responses in CA1 neurons was not altered in mice which underwent a sevoflurane anesthesia, which contrasts our recently published data with isoflurane. Although sevoflurane and isoflurane show a similar profile in view of their effects on voltage- and ligandgated ion channels [13], [14], [46]–[48], differences in e.g. the influence on the presynaptic glutamate release/reuptake [49] or on protein kinase C activity [50] have been reported. As protein kinase C activation is crucially involved in synaptic plasticity (for review see e.g. [51]), these differences might explain the discrepancy between isoflurane- and sevoflurane-induced medium-term effects on LTP.

In summary, in the present study, we show that sevoflurane anesthesia does not impair cognitive performance and CA1 hippocampal LTP formation in mice. Cognitive performance was even improved, which could be explained by the elevation of NR2B subunit expression in the hippocampus. Our data rather indicate little or no role of sevoflurane anesthesia in contributing to the development of cognitive impairment after anesthesia.
